# Sleep duration and quality in relation to chronic kidney disease and glomerular hyperfiltration in healthy men and women

**DOI:** 10.1371/journal.pone.0175298

**Published:** 2017-04-19

**Authors:** Chan-Won Kim, Yoosoo Chang, Eunju Sung, Kyung Eun Yun, Hyun-Suk Jung, Byung-Joon Ko, Min-Jung Kwon, Young Youl Hyun, Kyu-Beck Lee, Hyang Kim, Hocheol Shin, Seungho Ryu

**Affiliations:** 1 Center for Cohort Studies, Total Healthcare Center, Kangbuk Samsung Hospital, Sungkyunkwan University School of Medicine, Seoul, Republic of Korea; 2 Department of Occupational and Environmental Medicine, Kangbuk Samsung Hospital, Sungkyunkwan University School of Medicine, Seoul, Republic of Korea; 3 Department of Clinical Research Design & Evaluation, SAIHST, Sungkyunkwan University, Seoul, Republic of Korea; 4 Department of Family Medicine, Kangbuk Samsung Hospital, Sungkyunkwan University School of Medicine, Seoul, Republic of Korea; 5 Department of Laboratory Medicine, Kangbuk Samsung Hospital, Sungkyunkwan University School of Medicine, Seoul, Republic of Korea; 6 Division of Nephrology, Department of Internal Medicine, Kangbuk Samsung Hospital, Sungkyunkwan University School of Medicine, Seoul, Republic of Korea; University of Colorado Denver School of Medicine, UNITED STATES

## Abstract

**Background:**

It is unclear whether sleep duration and quality are associated with chronic kidney disease (CKD) and glomerular hyperfiltration. The aim of this study was to examine the association of sleep duration and quality with CKD and glomerular hyperfiltration in young and middle-aged adults.

**Methods:**

We conducted a cross-sectional study of men and women who underwent a health checkup examination, including assessment of sleep duration and quality (*n* = 241,607). Chronic kidney disease (CKD) was defined as an estimated glomerular filtration rate (eGFR) less than 60 ml/min/1.73 m^2^, and glomerular hyperfiltration was defined as eGFR above the age-/sex-specific 95^th^ percentile.

**Results:**

In a multinomial logistic regression analysis adjusting for relevant confounders, the adjusted prevalence ratios for CKD (95% confidence interval) comparing sleep durations of ≤ 5, 6, 8, and 9 hours with 7 hours were 1.22 (0.95–1.55), 0.93 (0.75–1.14), 0.97 (0.75–1.26), and 1.56 (1.06–2.30) in men and 0.98 (0.68–1.43), 1.03 (0.72–1.46), 1.39 (0.97–2.00), and 1.31 (0.78–2.22) in women, respectively. The corresponding prevalence ratios (95% confidence interval) for glomerular hyperfiltration were 1.00 (0.93–1.08), 0.97 (0.91–1.03), 1.03 (0.94–1.13), and 1.39 (1.13–1.72) in men and 1.04 (0.95–1.14), 0.96 (0.90–1.04), 1.11 (1.02–1.20), and 1.28 (1.14–1.45) in women, respectively. Poor subjective sleep quality was associated with glomerular hyperfiltration in men and women.

**Conclusion:**

In this large study of young and middle-aged adults, we found that long sleep duration was associated with CKD and glomerular hyperfiltration. Additionally, poor subjective sleep quality was associated with increased prevalence of glomerular hyperfiltration, suggesting the importance of adequate quantity and quality of sleep for kidney function.

## Introduction

Chronic kidney disease (CKD) is a worldwide public health problem that is increasing in incidence and prevalence [1]. Outcomes of CKD include not only progression to kidney failure, but also complications of reduced kidney function and increased risk of cardiovascular (CV) events, CV mortality, and all-cause mortality [2,3]. Additionally, previous studies have shown that estimated glomerular filtration rate (eGFR) has a U-shaped relationship with all-cause mortality, indicating the importance of both high and low eGFR [4,5]. Indeed, there is growing evidence that glomerular hyperfiltration is considered a marker of early renal damage [6]. In addition, several epidemiological studies have shown that both CKD and glomerular hyperfiltration are associated with subclinical measures of CV disease, even in an asymptomatic healthy population [7,8]. Thus, it is important to develop strategies to identify high-risk individuals before they develop CKD or glomerular hyperfiltration and to identify potentially modifiable risk factors.

Sleep of adequate quantity and quality is essential for homeostatic regulation [9], and some epidemiological studies have suggested that excess sleep is associated with CV disease and metabolic diseases such as obesity, diabetes, and hypertension, all of which are risk factors of CKD [10–13]. Thus, it could be speculated that excess sleep may be linked to CKD, but further studies are needed to evaluate whether this association is independent of comorbidities and relevant confounders, such as obesity, depression, or unhealthy behaviors including physical inactivity or high caloric intake. To date, previous studies exploring the association between sleep duration and the decline of kidney function have had inconsistent results, and the limited sample size has precluded the evaluation of the dose-response relationship [14]. In addition, most studies in this area have combined participants with normal and high eGFR to form the reference group. Subjective sleep quality may also be another important predictor of kidney function as optimal sleep duration varies by individual, but little data exists on the association of subjective sleep quality with CKD. Moreover, few studies have investigated the associations between sleep duration and quality and glomerular hyperfiltration [15]. The present study hypothesizes that extreme sleep duration and/or poor quality of sleep are associated with CKD and glomerular hyperfiltration in a large cohort of men and women participating in a health screening examination program.

## Subjects and methods

### Subjects

The Kangbuk Samsung Health Study is a cohort study of Korean men and women who underwent a comprehensive annual or biennial examination at the Kangbuk Samsung Hospital Screening Centers in Seoul and Suwon, South Korea [16,17]. More than 80% of participants were employees of various companies and local governmental organizations, together with their spouses. In South Korea, the Industrial Safety and Health Law requires annual or biennial health screening exams of all employees, offered free of charge. The remaining participants voluntarily purchased screening exams. The study population of the present study consisted of 275,811 men and women who completed a sleep assessment questionnaire between March 2011 and December 2014. This study was approved by the Institutional Review Board of Kangbuk Samsung Hospital. The informed consent requirement for this study was waived by the Institutional Review Board because researchers only retrospectively accessed a de-identified database for analysis purposes.

We excluded 32,204 participants for the following reasons: a history of sleep-related problems such as narcolepsy (*n* = 190) or obstructive sleep apnea (*n* = 1,202), a history of cancer (*n* = 8,143), and participants who worked a night shift (*n* = 19,478). We further excluded participants with missing data for eGFR or proteinuria (*n* = 8,267). Because some individuals met more than one exclusion criterion, the total number of eligible participants for this study was 241,607.

### Assessment of sleep duration and quality

Sleep duration and quality were assessed using the Pittsburgh Sleep Quality Index (PSQI) during the health screening exam [18]. The PSQI is a 19-item validated self-administered questionnaire that assesses habitual sleep during the past month. The PSQI is composed of seven components: subjective sleep quality, sleep latency, sleep duration, habitual sleep efficiency, sleep disturbances, use of sleeping medication, and daytime function. The Korean version of the PSQI was used in this study and has been validated for use in Korea [19]. Sleep duration and subjective sleep quality were derived from this index, and sleep duration was rounded to the nearest hour and categorized as ≤ 5, 6, 7, 8, or ≥ 9 hours. Regarding subjective sleep quality, the response categories of the PSQI were very good, fairly good, fairly bad, and very bad. The last two categories were defined as poor subjective sleep quality.

### Assessment of other variables

Demographic characteristics, smoking status, alcohol consumption, marital status, medical history, and medication use were collected through standardized, self-administered questionnaires. Smoking status was categorized into never, former, or current smoker, and alcohol consumption was categorized into none, moderate (≤ 20 g/day), or high intake (> 20 g/day). Physical activity levels were determined by the Korean-validated version of the short form of the International Physical Activity Questionnaire (IPAQ) and were classified into three categories: inactive, minimally active, and health-enhancing physically active (HEPA). Previous history of CV disease was defined as history of ischemic heart disease or cerebrovascular disease. Body weight was measured with light clothing and without shoes to the nearest 0.1 kilogram using a digital scale, and height was measured by trained nurses. Body mass index (BMI) was calculated as weight/height squared (kg/m^2^) and was classified according to Asian-specific criteria (underweight, BMI < 18.5 kg/m^2^; normal weight, BMI of 18.5 to < 23 kg/m^2^; overweight, BMI of 23 to 24.9 kg/m^2^; and obese, BMI ≥ 25 kg/m^2^). Depression was assessed using the Korean version [20] of the Center for Epidemiologic Studies Depression (CES-D) Scale [21], and clinically significant depression was defined when the CES-D score was 16 or higher.

Usual dietary intake was assessed using a 103-item, self-administered food frequency questionnaire (FFQ) designed and validated for use in Korea [22]. The validity and reproducibility of our FFQ were evaluated previously by comparing nutrient and food intake derived from 12 24-hour dietary recalls during four seasons and with a second FFQ administered one year later [22]. Total energy and nutrient intake were calculated using a food composition table developed by the Korean Nutrition Society.

Blood samples were collected following a fast of at least 10 hours. The methods for measuring serum levels of total cholesterol, low-density lipoprotein (LDL) cholesterol, triglycerides, high-density lipoprotein (HDL) cholesterol levels, insulin, and high-sensitivity C-reactive protein (hsCRP) have been reported elsewhere [23]. Serum creatinine values were measured with the kinetic alkaline picrate (Jaffe) method traceable to isotope-dilution mass spectroscopy. The within-batch and total coefficients of variation for creatinine determinations were 1.4–3.9% for the duration of the study. We calculated eGFR using the Chronic Kidney Disease Epidemiology Collaboration (CKD-EPI) equation. Chronic kidney disease (CKD) was defined as eGFR < 60 ml/min per 1.73 m^2^, and glomerular hyperfiltration was defined as eGFR above the age-/sex-specific 95^th^ percentile [24,25]. Urine protein was measured semi-quantitatively by urine dipstick (URiSCAN Urine test strips, YD Diagnostics) assessment on fresh, midstream urine samples and was reported in the following six grades: absent, trace, 1+, 2+, 3+, and 4+ (corresponding to protein levels of undetectable, 10 mg/dL, 30 mg/dL, 100 mg/dL, 300 mg/dL, and 1000 mg/dL, respectively). Proteinuria was defined as a grade of 1+ or greater. Insulin resistance was assessed with the homeostasis model assessment of insulin resistance (HOMA-IR) as fasting glucose (mg/dL) × fasting insulin (µIU/mL)/405. Hypertension was defined as a systolic blood pressure ≥ 140 mmHg, diastolic blood pressure ≥ 90 mmHg, or the current use of antihypertensive medication. Diabetes was defined as a fasting serum glucose ≥ 126 mg/dl, hemoglobin A1c ≥ 6.5%, or the current use of antidiabetic medication.

### Statistical analysis

Characteristics of the study participants were explored according to sleep duration category. To test for linear trends, category numbers were used as continuous variables in regression models. To evaluate the association of CKD and glomerular hyperfiltration across categories of sleep duration and sleep quality, multinomial logistic regression models were used to estimate prevalence ratios and 95 percent confidence intervals (95% CIs). We used three models with progressively more adjustment for confounding variables. Multivariable model 1 was adjusted for age, sex, study center (Seoul or Suwon), year of screening exam (1-year categories), smoking status (never, past, current, or unknown), alcohol intake (0, < 20, ≥ 20g/d, or unknown), level of education, marital status, depression score, and total calorie intake (in quintile or missing). Model 2 included all of the variables that were used in model 1 plus history of diabetes, history of hypertension, and history of CV disease. To assess whether the associations of sleep duration and quality with CKD and glomerular hyperfiltration were mediated by BMI, glucose, systolic blood pressure, HOMA-IR, or hsCRP, we included these variables in multivariable models. To obtain the P-trend, we conducted tests for linear trend by assigning the median value to each category of sleep duration and modeling this value as a continuous variable. Tests for a quadratic trend were conducted by assigning the median value of each sleep duration category and including the squared terms centered at a 7- hour sleep duration. Additionally, we conducted a sensitivity analysis in which we repeated our analysis after excluding participants with history of CV disease because of the possibility that they could be individuals with comorbidities associated with extreme sleep duration or poor sleep quality. All P-values were two-tailed, and values of *P* < 0.05 were considered statistically significant. For data analysis, we used STATA version 14.0 (StataCorp, College Station, TX, USA).

## Results

Table 1 and Table 2 present the baseline characteristics of men and women in our study, respectively. The respective mean age of men and women was 41.0 and 40.2 years. The prevalence of CKD and glomerular hyperfiltration were 1.8% and 4.8%, respectively. The mean sleep duration was 6.5 hours, and 18.3% of participants reported poor sleep quality. Compared with persons who slept 7 hours, both men and women who slept ≤ 5 hours and ≥ 9 hours showed similar features and patterns in socioeconomic status, behaviors, and comorbidities; they were less likely to be married, more likely to be current smokers and drinkers, and more likely to have depression, hypertension, diabetes, a history of cardiovascular disease, and poor subjective sleep quality. Men and women with long sleep duration (≥ 9 hours) had higher levels of the components or traits of metabolic syndrome, such as blood pressure, triglycerides, and HOMA-IR, and lower levels of HDL cholesterol compared with those with a 7-hour sleep duration. These trends were also observed in sleep-deprived women, but not in sleep- deprived men.

**Table 1:** Baseline characteristics of study participants by sleep duration among men.

Characteristics	Overall	Sleep duration (hours)					*P* for quadratic trend
		≤ 5	6	7	8	≥ 9	
**Number**	135,633	21,081	55,249	45,242	12,533	1,528	
**Age (years)**^a^	41.0 (9.0)	40.7 (8.9)	40.6 (8.2)	41.0 (9.0)	42.5 (10.9)	46.2 (14.3)	<0.001
**Seoul center (%)**	66.3	72.0	67.5	63.0	63.0	65.8	<0.001
**Obesity (%)**	39.4	44.5	40.0	37.2	36.1	36.8	<0.001
**Current smoker (%)**	38.6	42.8	38.4	37.3	37.2	39.0	0.136
**Alcohol intake (%)**^b^	36.7	40.8	36.9	34.8	35.2	39.0	0.119
**HEPA (%)**	17.6	18.0	17.3	17.4	18.6	20.6	<0.001
**High education (%)**^d^	86.8	87.0	89.2	87.0	78.8	58.5	<0.001
**Married (%)**	81.0	76.1	80.9	82.7	83.6	78.5	<0.001
**Depression (%)**	7.8	12.9	7.4	6.1	6.6	11.4	0.196
**Hypertension**	16.5	17.3	16.0	16.0	18.2	24.4	<0.001
**Diabetes**	6.1	6.8	5.5	5.8	7.4	12.8	<0.001
**History of CVD**	1.6	1.7	1.4	1.5	2.2	4.1	<0.001
**BMI (kg/m**^**2**^**)**	24.5 (2.9)	24.8 (3.0)	24.5 (2.9)	24.3 (3.0)	24.2 (3.0)	24.2 (3.1)	<0.001
**Systolic BP (mmHg)**^a^	114.6 (11.7)	114.7 (11.8)	114.4 (11.6)	114.5 (11.7)	115.0 (12.0)	116.3 (12.8)	<0.001
**Diastolic BP (mmHg)**^a^	74.0 (9.4)	74.0 (9.7)	73.9 (9.4)	74.0 (9.4)	74.2 (9.3)	74.8 (9.9)	<0.001
**Glucose (mg/dl)**^a^	98.4 (16.5)	98.7 (18.1)	98.1 (15.8)	98.4 (16.4)	99.2 (16.6)	101.9 (22.0)	<0.001
**Total cholesterol (mg/dl)**^a^	200.0 (34.6)	200.6 (35.2)	200.2 (34.4)	199.6 (34.2)	199.6 (35.5)	198.8 (36.1)	0.028
**LDL-C (mg/dl)**^a^	127.4 (31.4)	127.5 (31.7)	127.6 (31.3)	127.2 (31.2)	127.2 (32.0)	126.9 (32.5)	0.159
**HDL-C (mg/dl)**^a^	52.8 (12.7)	52.8 (12.9)	52.9 (12.7)	52.8 (12.7)	52.5 (12.8)	51.5 (12.8)	<0.001
**Triglycerides (mg/dl)**^c^	115 (81–165)	117 (82–169)	114 (81–194)	114 (81–163)	115 (81–165)	121 (84–176)	0.002
**ALT (U/l)**^c^	23 (17–34)	24 (17–35)	23 (17–34)	23 (17–34)	24 (17–34)	24 (17–35)	0.051
**HOMA-IR**^c^	1.28 (0.84–1.92)	1.29 (0.84–1.97)	1.27 (0.83–1.89)	1.28 (0.84–1.92)	1.32 (0.86–1.97)	1.35 (0.85–2.09)	<0.001
**hsCRP (mg/l)**^c^	0.5 (0.3–1.0)	0.6 (0.3–1.1)	0.5 (0.3–1.0)	0.5 (0.3–1.0)	0.5 (0.3–1.1)	0.6 (0.3–1.2)	<0.001
**Total energy intake**^c^^,^^e^	1667.5 (1348.6–2056.4)	1678.6 (1330.8–2093.3)	1667.9 (1353.7–2053.3)	1663.7 (1350.0–2042.7)	1661.3 (1345.8-	1673.2 (1344.9–2064.9)	0.368
**Poor sleep quality**	14.8	32.9	14.9	8.5	6.6	10.0	<0.001

Data are expressed as

^a^ mean (standard deviation)

^c^ median (interquartile range), or percentage.

^b^ ≥ 20 g of ethanol per day

^d^ ≥ college graduate

^e^ among 165,861 participants with plausible estimated energy intake levels (within three standard deviations from the log-transformed mean energy intake).

CVD, cardiovascular disease; ALT, alanine aminotransferase; BMI, body mass index; BP, blood pressure; HDL-C, high-density lipoprotein cholesterol; HEPA, health-enhancing physical activity; hsCRP, high sensitivity C-reactive protein; HOMA-IR, homeostasis model assessment of insulin resistance.

**Table 2:** Baseline characteristics of study participants by sleep duration among women.

Characteristics	Overall	Sleep duration (hours)					*P* for quadratic trend
		≤ 5	6	7	8	≥ 9	
**Number**	105,974	15,378	30,413	35,442	19,602	5,139	
**Age (years)**^a^	40.2 (9.7)	41.8 (11.0)	40.5 (9.6)	39.9 (9.2)	39.5 (9.5)	39.1 (10.8)	<0.001
**Seoul center (%)**	64.6	69.4	68.8	63.8	58.4	54.1	<0.001
**Obesity (%)**	13.5	17.3	14.0	12.0	12.2	13.4	<0.001
**Current smoker (%)**	2.5	3.7	2.6	2.0	2.2	2.3	0.022
**Alcohol intake (%)**^b^	5.9	8.6	6.0	5.0	5.2	6.0	0.041
**HEPA (%)**	15.0	16.2	15.3	15.0	14.2	12.5	<0.001
**High education (%)**^d^	74.3	68.4	74.6	76.6	75.0	71.4	0.006
**Married (%)**	82.4	76.6	79.4	84.2	87.5	87.0	<0.001
**Depression (%)**	16.3	27.5	17.4	12.9	12.6	14.9	<0.001
**Hypertension**	7.0	10.0	7.1	6.0	6.4	7.0	0.038
**Diabetes**	2.7	3.7	2.7	2.3	2.7	3.4	0.524
**History of CVD**	0.9	1.4	0.9	0.8	0.8	1.1	0.021
**BMI (kg/m**^**2**^**)**	21.8 (3.0)	22.2 (3.3)	21.9 (3.1)	21.6 (2.9)	21.6 (3.0)	21.6 (3.1)	<0.001
**Systolic BP (mmHg)**^a^	102.5 (12.2)	103.5 (12.8)	102.5 (12.3)	102.2 (12.1)	102.2 (12.0)	102.3 (12.0)	0.003
**Diastolic BP (mmHg)**^a^	65.5 (8.7)	66.1 (9.1)	65.5 (8.8)	65.3 (8.6)	65.2 (8.6)	65.2 (8.4)	<0.001
**Glucose (mg/dl)**^a^	92.3 (12.3)	92.5 (13.1)	92.2 (12.0)	92.0 (11.5)	92.4 (12.7)	92.5 (14.3)	0.013
**Total cholesterol (mg/dl)**^a^	189.0 (33.3)	191.7 (34.1)	189.6 (33.1)	188.5 (32.8)	187.6 (33.4)	186.9 (33.8)	<0.001
**LDL-C (mg/dl)**^a^	111.4 (30.4)	113.4 (31.7)	111.5 (30.4)	110.8 (29.8)	110.9 (30.3)	110.5 (30.5)	0.001
**HDL-C (mg/dl)**^a^	64.8 (14.8)	64.9 (15.3)	65.3 (14.6)	65.1 (14.6)	63.9 (14.4)	63.2 (14.4)	<0.001
**Triglycerides (mg/dl)**^c^	72 (55–99)	74 (56–103)	71 (55–98)	71 (55–96)	72 (56–99)	73 (56–102)	<0.001
**ALT (U/l)**^c^	13 (11–18)	14 (11–19)	13 (11–18)	13 (10–17)	13 (10–18)	13 (10–17)	<0.001
**HOMA-IR**^c^	1.06 (0.71–1.55)	1.04 (0.69–1.57)	1.04 (0.70–1.54)	1.06 (0.72–1.53)	1.08 (0.73–1.57)	1.10 (0.74–1.61)	<0.001
**hsCRP (mg/l)**^c^	0.3 (0.2–0.7)	0.3 (0.2–0.7)	0.3 (0.2–0.7)	0.3 (0.2–0.6)	0.3 (0.2–0.7)	0.3 (0.2–0.7)	<0.001
**Total energy intake**^c^^,^^e^	1437.5 (1092.2–1810.2)	1411.9 (1052.9–1808.1)	1417.7 (1074.4–1793.4)	1443.3 (1104.3–1806.0)	1465.1 (1124.0–1824.4)	1476.4 (1107.6–1861.2)	<0.001
**Poor sleep quality**	22.8	53.8	25.5	14.8	11.4	13.9	<0.001

Data are expressed as

^a^ mean (standard deviation)

^c^ median (interquartile range), or percentage.

^b^ ≥ 20 g of ethanol per day

^d^ ≥ college graduate

^e^ among 165,861 participants with plausible estimated energy intake levels (within three standard deviations from the log-transformed mean energy intake).

CVD, cardiovascular disease; ALT, alanine aminotransferase; BMI, body mass index; BP, blood pressure; HDL-C, high-density lipoprotein cholesterol; HEPA, health-enhancing physical activity; hsCRP, high sensitivity C-reactive protein; HOMA-IR, homeostasis model assessment of insulin resistance.

The proportion of participants with CKD among those who slept ≤ 5, 6, 7, 8, and ≥ 9 hours was 0.5, 0.3, 0.4, 0.5, and 0.9%, respectively. The proportion of participants with glomerular hyperfiltration among those who slept ≤ 5, 6, 7, 8, and ≥ 9 hours was 4.9, 4.6, 4.8, 5.4, and 6.7%, respectively. Results from multinomial logistic regression analyses adjusted for age, sex, center, year of screening exam, smoking status, alcohol intake, physical activity, marital status, level of education, depression, total calorie intake, and history of comorbid conditions are presented in Table 3 and Table 4. Short sleep duration was not significantly associated with CKD in men or women. Long sleep duration (≥ 9 hours) was significantly associated with CKD in men, but not in women. The adjusted prevalence ratios (95% CIs) for CKD comparing a sleep duration ≥ 9 hours with that of 7 hours were 1.56 (1.06–2.30) and 1.31 (0.78–2.22) in men and women, respectively. Significant increases in the risk of glomerular hyperfiltration existed among both men and women who slept 9 hours, compared with those who slept 7 hours in fully adjusted models. The adjusted prevalence ratios (95% CIs) for glomerular hyperfiltration comparing a sleep duration ≥ 9 hours with that of 7 hours were 1.39 (1.13–1.72) and 1.28 (1.14–1.45) in men and women, respectively. In dose-response analysis, sleep duration largely showed J-shaped association with CKD and glomerular hyperfiltration with the lowest risk at a sleep duration of 6–7 hours (S1–S4 Figs). However, the association of short sleep duration with CKD and glomerular hyperfiltration did not reach significant levels after adjustment for relevant confounders.

**Table 3:** Prevalence ratios^a^ (95% CI) of CKD and glomerular hyperfiltration by sleep duration and subjective sleep quality among men.

	Sleep duration (hours)					*P* for quadratic trend	Subjective sleep quality	
	≤5	6	7	8	≥9		Good	Poor
**Number**	21,081	55,249	45,242	12,533	1,528		115,623	20,010
**CKD**								
**Cases (%)**	113 (0.5)	187 (0.3)	210 (0.5)	94 (0.8)	40 (2.6)		535 (0.5)	109 (0.5)
**Crude**	1.16 (0.92–1.46)	0.73 (0.60–0.89)	Reference	1.62 (1.27–2.07)	5.88 (4.18–8.29)	<0.001	Reference	1.19 (0.97–1.46)
**Multivariate-adjusted PRs**^a^								
**Model 1**	1.23 (0.97–1.56)	0.93 (0.76–1.14)	Reference	0.98 (0.76–1.27)	1.52 (0.76–1.13)	0.062	Reference	1.09 (0.87–1.36)
**Model 2**	1.22 (0.95–1.55)	0.93 (0.75–1.14)	Reference	0.97 (0.75–1.26)	1.56 (1.06–2.30)	0.059	Reference	1.06 (0.84–1.32)
**Hyperfiltration**								
**Cases (%)**	1,054 (5.0)	2,571 (4.7)	2,184 (4.8)	624 (5.0)	101 (6.6)		5,449 (4.7)	1,085 (5.4)
**Crude**	1.04 (0.96–1.12)	0.96 (0.91–1.02)	Reference	1.04 (0.95–1.14)	1.43 (1.16–1.76)	<0.001	Reference	1.16 (1.09–1.24)
**Multivariate-adjusted PRs**^a^								
**Model 1**	1.01 (0.93–1.09)	0.97 (0.91–1.02)	Reference	1.03 (0.94–1.13)	1.41 (1.14–1.74)	0.001	Reference	1.10 (1.03–1.18)
**Model 2**	1.00 (0.93–1.08)	0.97 (0.91–1.03)	Reference	1.03 (0.94–1.13)	1.39 (1.13–1.72)	0.002	Reference	1.09 (1.02–1.17)

^a^ Estimated from multinomial logistic regression models. Multivariable model 1 was adjusted for age, center, year of screening exam, smoking status, alcohol intake, physical activity, marital status, education level, total caloric intake, and depression; model 2 includes all of the variables from model 1 plus adjustment for history of diabetes, history of hypertension, and history of cardiovascular disease.

CKD, chronic kidney disease; BMI, body mass index; CI, confidence intervals; PR, prevalence ratio. CKD is defined as GFR < 60 ml/min per 1.73 m^2^.

**Table 4:** Prevalence ratios^a^ (95% CI) of CKD and glomerular hyperfiltration by sleep duration and subjective sleep quality among women.

	Sleep duration (hours)					*P* for quadratic trend	Subjective sleep quality	
	≤5	6	7	8	≥9		Good	Poor
**Number**	15,378	30,413	35,442	19,602	5,139		81.782	24,192
**CKD**								
**Cases (%)**	54 (0.4)	64 (0.2)	69 (0.2)	58 (0.3)	21 (0.4)		188 (0.2)	78 (0.3)
**Crude**	1.80 (1.26–2.58)	1.08 (0.77–1.51)	Reference	1.53 (1.08–2.18)	2.15 (1.32–3.50)	0.002	Reference	1.41 (1.09–1.84)
**Multivariate-adjusted PRs**^a^								
**Model 1**	1.06 (0.73–1.54)	1.02 (0.72–1.44)	Reference	1.46 (1.02–2.09)	1.31 (0.78–2.19)	0.069	Reference	1.23 (0.93–1.63)
**Model 2**	0.98 (0.68–1.43)	1.03 (0.72–1.46)	Reference	1.39 (0.97–2.00)	1.31 (0.78–2.22)	0.080	Reference	1.14 (0.86–1.52)
**Hyperfiltration**								
**Cases (%)**	729 (4.7)	1,364 (4.5)	1,725 (4.9)	1,105(5.6)	348 (6.8)		3,939 (4.8)	1,332 (5.5)
**Crude**	0.97 (0.89–1.06)	0.92 (0.85–0.99)	Reference	1.17 (1.08–1.26)	1.42 (1.26–1.60)	<0.001	Reference	1.15 (1.08–1.23)
**Multivariate-adjusted PRs**^a^								
**Model 1**	1.05 (0.95–1.14)	0.97 (0.90–1.44)	Reference	1.11 (1.03–1.20)	1.29 (1.14–1.45)	<0.001	Reference	1.13 (1.06–1.21)
**Model 2**	1.04 (0.95–1.14)	0.96 (0.90–1.04)	Reference	1.11 (1.02–1.20)	1.28 (1.14–1.45)	<0.001	Reference	1.13 (1.06–1.21)

^a^ Estimated from multinomial logistic regression models. Multivariable model 1 was adjusted for age, center, year of screening exam, smoking status, alcohol intake, physical activity, marital status, education level, total caloric intake, and depression; model 2 includes all of the variables from model 1 plus adjustment for history of diabetes, history of hypertension, and history of cardiovascular disease.

CKD, chronic kidney disease; BMI, body mass index; CI, confidence intervals; PR, prevalence ratio. CKD is defined as GFR < 60 ml/min per 1.73 m^2^.

We further examined the association of sleep duration and quality with proteinuria. While there was no significant association between short sleep duration and CKD, short sleep duration (≤5 hours) was associated with proteinuria in women, but not in men. Conversely, long sleep duration was associated with proteinuria in men but not in women (S3 and S4 Tables). Poor subjective sleep quality was significantly associated with glomerular hyperfiltration in both men and women, but was not associated with CKD.

To investigate whether the increased prevalence of CKD and glomerular hyperfiltration was mediated by potential biological mediators related to extreme duration or poor quality of sleep, we conducted further adjustment for the potential mediators of BMI, glucose, systolic blood pressure, HOMA-IR, and level of hsCRP (S1 and S2 Tables). However, these adjustments did not appreciably change the results. In a sensitivity analysis, our results did not qualitatively change after further excluding participants with history of CV disease.

## Discussion

In this large study of young and middle-aged men and women, we found that long sleep duration was associated with both CKD and glomerular hyperfiltration, independent of age, sex, marital status, education level, lifestyle factors, total calorie intake, and comorbid conditions such as hypertension, diabetes, and cardiovascular disease. Additionally, poor subjective sleep quality was independently associated with the increased prevalence of glomerular hyperfiltration. The large sample size of our study allowed for the detailed dose-response analysis of the association of sleep duration with CKD and glomerular hyperfiltration.

Previous studies evaluating the association between sleep duration and kidney dysfunction have yielded inconsistent results. One population-based study of Japanese middle-aged adults showed that sleeping less than 5 hours per night is not associated with CKD, and another cross-sectional analysis of the Swiss general population displayed a linear relationship between objectively measured sleep duration and eGFR. In those studies, sleep duration was not classified into long sleep duration [15,26]. In a cross-sectional study of elderly Chinese patients with hypertension, short sleep duration was associated with CKD [27]. Available evidence from two longitudinal studies that have evaluated this association remains inconclusive. In a prospective cohort study of 4,238 participants from the Nurses’ Health Study, participants with a short sleep duration (5 hours or less) were shown to be 79% more likely to develop rapid decline in renal function (a decrease in eGFR ≥ 30%) in comparison with participants reporting a sleep duration of 7 hours [28]. However, no association between renal function and sleep duration was observed in a cohort study conducted in Japan [29]. Based on sample size, these studies were also underpowered to assess the dose-response relationship. We found only one study that has investigated the association between sleep duration and glomerular hyperfiltration using a nationally representative sample in the United States [30]. In that study, the association differed by race-ethnicity, and long sleep duration was significantly related to glomerular hyperfiltration among Mexican Americans. Consistent with the findings of that study, we found that long sleep duration was associated with glomerular hyperfiltration.

We found that the association between long sleep duration and CKD appeared to be stronger in men than in women. In contrast to the relevant literature, which reported that middle-aged women exhibit a higher prevalence of CKD than middle-aged men [31], our results showed a higher prevalence of CKD in men than in women. However, due to the possibility that these findings arose by chance in sex-stratified analyses, further studies are needed to clarify whether the effects of long sleep duration on kidney function are sex-specific.

Consistent with a recent meta-analysis of three observational studies, we found that short sleep duration was associated with an increased prevalence of proteinuria in women, but not in men.[14] Conversely, only men reporting long sleep duration had an increased prevalence of proteinuria. As suggested in previous literature, these differences may be related to sex-based variation in the impact of sleep duration on health outcomes, with a higher risk of metabolic disturbances relevant to proteinuria among sleep-deprived women compared with men [16,32]. However, these relationships have not been clarified regarding the development of proteinuria. Future studies are needed to assess the effects of extreme sleep duration on proteinuria and whether these effects differ by sex.

With respect to the association between subjective sleep quality and kidney dysfunction, the prevalence of poor subjective sleep quality appeared to have a non-linear relationship with eGFR measures in one recent cross-sectional study, but the interpretation is limited by the small sample size of the study [15]. Thus, there is need for further studies to assess whether poor subjective sleep quality would be related to both lower and higher eGFR and our findings show that poor subjective sleep quality is significantly associated with increased prevalence of glomerular hyperfiltration, but is not associated with CKD, suggesting that poor subjective sleep quality might be related to earlier kidney dysfunction.

The mechanisms underlying the association between long sleep duration and kidney dysfunction are not clear. Evidence is emerging that long sleep duration is associated with subclinical inflammation and increased arterial stiffness, which can induce progressive decline in renal function [33–36]. Sleep fragmentation and concomitant higher sympathetic tone, to which long sleep duration is likely attributable, have evolved multiple pathways to induce renal function loss via the release of catecholamine, dysregulation of the renin-angiotensin system, and atherogenesis [37–40]. Physical inactivity related to long sleep disturbance may explain the association of sleep duration with CKD and glomerular hyperfiltration [41–43]. In the present analysis, however, adjusting for physical activity did not attenuate this association, suggesting that other pathways may be implicated in the association between sleep duration and renal function. Quantitatively or qualitatively insufficient sleep has reported associations with obesity, decreased insulin sensitivity, diabetes, and hypertension, all of which can induce kidney dysfunction via abnormal production of adipokines and hormones, hemodynamic changes such as increased filtration fraction, and consequent glomerular hypertension in remnant nephrons[44–47]. In the crude model of our study, short sleep duration was associated with CKD, but this association was attenuated by relevant confounders or comorbidities, suggesting that the variance between short sleep duration and kidney dysfunction may be partially explained by aging, depression, or unhealthy behaviors, such as physical inactivity or an increase in caloric intake.

Several limitations of this study should be taken into consideration. First, the categorization of sleep duration may be misclassified insofar as the self-reported assessment of sleep duration might be only modestly correlated with objectively measured sleep duration. Although this self-reported assessment was conducted using the validated structured PSQI questionnaire [48], future studies are needed to determine the dose-response relationship between objectively measured sleep duration and renal dysfunction. Second, the associations between sleep duration and markers of kidney dysfunction may be partially confounded by obstructive sleep apnea [49]. Although our exclusion criteria included prior diagnosis of sleep apnea, we did not screen participants for its presence. Alternatively, adjustment for BMI and poor subjective sleep quality highly correlated with obstructive sleep apnea [50] did not materially change the association of sleep duration with CKD and glomerular hyperfiltration in this study. Third, the cross-sectional design of our study limited our ability to establish temporal relationships and to infer causality. Nevertheless, all studies were performed after participants had completed their health exam, and the estimates did not change after excluding participants with history of CV disease, thereby minimizing the risk of reverse causation. Fourth, CKD and glomerular hyperfiltration were identified by a single measurement. Finally, most subjects in this study were highly educated, young or middle-aged Korean men and women who regularly attended health screening exams. Accordingly, caution should be used when generalizing our results to other populations.

A major strength of our study is the large sample size allowing for comprehensive analysis of the relationships of sleep duration with CKD and glomerular hyperfiltration when considering both extreme sleep duration and sleep quality. In addition, our results are strengthened by a detailed panel of potential confounders and mediators that were collected with high-quality data measurement procedures, as well as the use of a relatively healthy study population that was less likely to be affected by comorbidities.

In summary, our results showed that long sleep duration was associated with CKD and glomerular hyperfiltration. Additionally we also found that poor subjective sleep quality was associated with an increased prevalence of glomerular hyperfiltration. Future studies are needed to evaluate whether there is a causal relationship between long duration of sleep and the development of CKD and glomerular hyperfiltration.

## Supporting information

S1 Fig Multivariable-adjusted odds ratios for CKD by sleep duration among men. (TIF)

S2 Fig Multivariable-adjusted odds ratios for hypperfiltration by sleep duration among men. (TIF)

S3 Fig Multivariable-adjusted odds ratios for CKD by sleep duration among women. (TIF)

S4 Fig Multivariable-adjusted odds ratios for hypperfiltration by sleep duration among women. (TIF)

S1 Table Mediation analysis of the associations of sleep duration and subjective sleep quality with CKD and glomerular hyperfiltration among men. ^a^ Estimated from multinomial logistic regression models. Multivariable model 1 was adjusted for age, center, year of screening exam, smoking status, alcohol intake, physical activity, marital status, education level, total calorie intake, depression, history of diabetes, history of hypertension, history of cardiovascular disease, and BMI; model 2 includes all of the variables from model 1 plus adjustment for glucose instead of BMI; model 3 includes all of the variables from model 1 plus adjustment for systolic blood pressure instead of BMI; model 4 includes all of the variables from model 1 plus adjustment for HOMA-IR instead of BMI; and model 5 includes all of the variables from model 1 plus adjustment for hsCRP instead of BMI.
CKD, chronic kidney disease; BMI, body mass index; CI, confidence intervals; PR, prevalence ratio.
CKD is defined as GFR < 60 ml/min per 1.73 m^2^
(DOCX)

S2 Table Mediation analysis of the associations of sleep duration and subjective sleep quality with CKD and glomerular hyperfiltration among women. ^a^ Estimated from multinomial logistic regression models. Multivariable model 1 was adjusted for age, center, year of screening exam, smoking status, alcohol intake, physical activity, marital status, education level, total calorie intake, depression, history of diabetes, history of hypertension, history of cardiovascular disease, and BMI; model 2 includes all of the variables from model 1 plus adjustment for glucose instead of BMI; model 3 includes all of the variables from model 1 plus adjustment for systolic blood pressure instead of BMI; model 4 includes all of the variables from model 1 plus adjustment for HOMA-IR instead of BMI; and model 5 includes all of the variables from model 1 plus adjustment for hsCRP instead of BMI.
CKD, chronic kidney disease; BMI, body mass index; CI, confidence intervals; PR, prevalence ratio.
CKD is defined as GFR < 60 ml/min per 1.73 m^2^
(DOCX)

S3 Table Odds ratiosa (95% CI) of proteinuria according to sleep duration and subjective sleep quality among men. ^a^ Estimated from multinomial logistic regression models. Multivariable model 1 was adjusted for age, center, year of screening exam, smoking status, alcohol intake, physical activity, marital status, education level, total caloric intake, and depression; model 2 includes all of the variables from model 1 plus adjustment for history of diabetes, history of hypertension, and history of cardiovascular disease.
CKD, chronic kidney disease; BMI, body mass index; CI, confidence intervals; PR, prevalence ratio.
CKD is defined as GFR < 60 ml/min per 1.73 m^2^
(DOCX)

S4 Table Odds ratiosa (95% CI) of proteinuria according to sleep duration and subjective sleep quality among women. ^a^ Estimated from multinomial logistic regression models. Multivariable model 1 was adjusted for age, center, year of screening exam, smoking status, alcohol intake, physical activity, marital status, education level, total caloric intake, and depression; model 2 includes all of the variables from model 1 plus adjustment for history of diabetes, history of hypertension, and history of cardiovascular disease.
CKD, chronic kidney disease; BMI, body mass index; CI, confidence intervals; PR, prevalence ratio.
CKD is defined as GFR < 60 ml/min per 1.73 m^2^.
(DOCX)
