# Supplementary figures and images for: Sleep duration and quality in relation to chronic kidney disease and glomerular hyperfiltration in healthy men and women
Source: PLoS One. 2017 Apr 19;12(4):e0175298. doi: 10.1371/journal.pone.0175298 (PMC5396878; doi:10.1371/journal.pone.0175298)

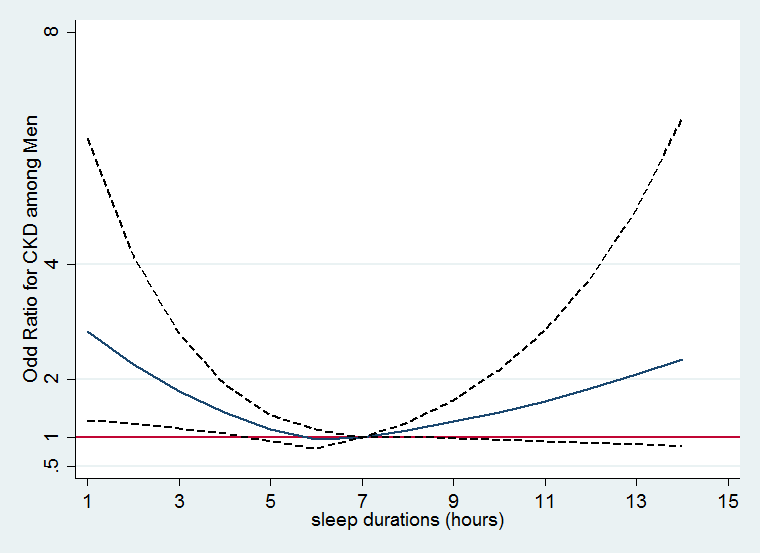

Supplement: S1 Fig — (TIF) [file pone.0175298.s001.tif]

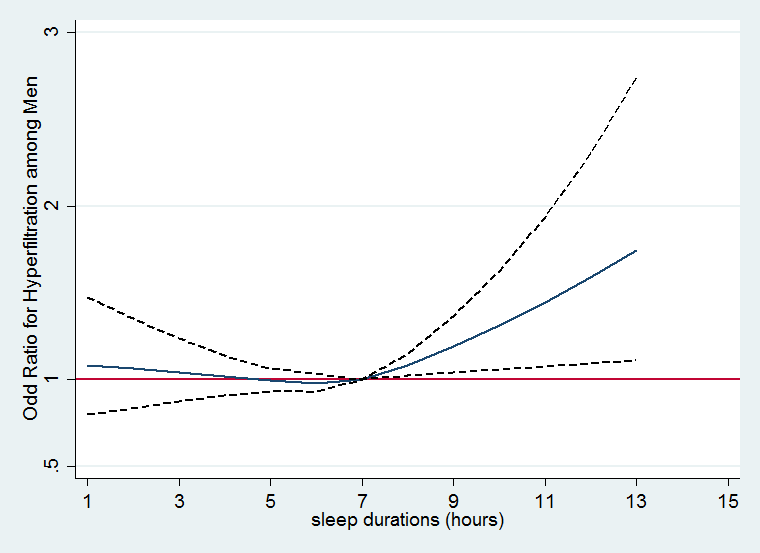

Supplement: S2 Fig — (TIF) [file pone.0175298.s002.tif]

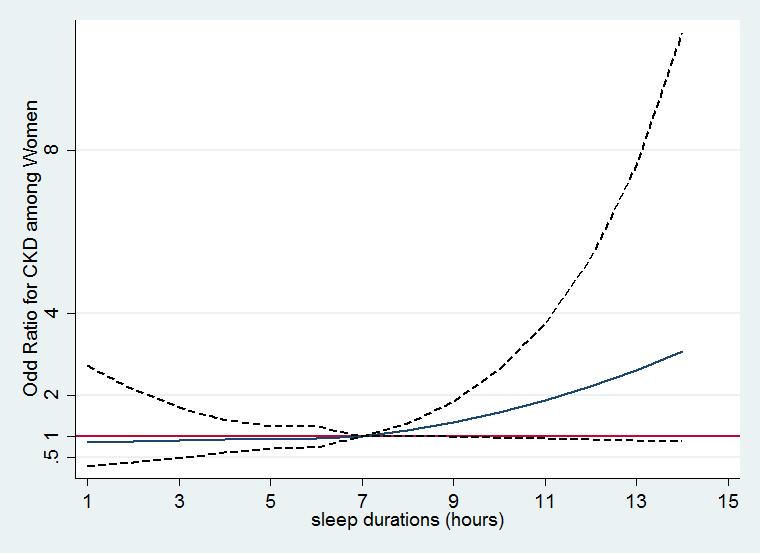

Supplement: S3 Fig — (TIF) [file pone.0175298.s003.tif]

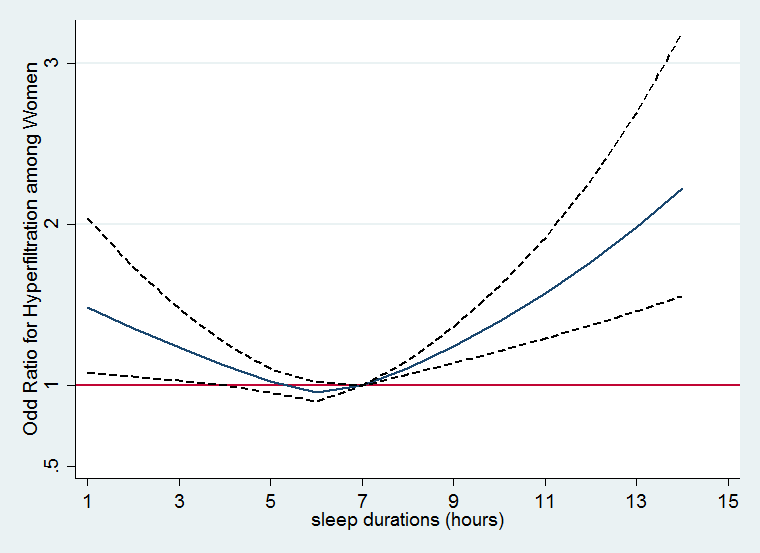

Supplement: S4 Fig — (TIF) [file pone.0175298.s004.tif]
