# Supplementary material for: Sleep duration and quality in relation to chronic kidney disease and glomerular hyperfiltration in healthy men and women
Source: PLoS One. 2017 Apr 19;12(4):e0175298. doi: 10.1371/journal.pone.0175298 (PMC5396878; doi:10.1371/journal.pone.0175298)
Supplement: S1 Table — a Estimated from multinomial logistic regression models. Multivariable model 1 was adjusted for age, center, year of screening exam, smoking status, alcohol intake, physical activity, marital status, education level, total calorie intake, depression, history of diabetes, history of hypertension, history of cardiovascular disease, and BMI; model 2 includes all of the variables from model 1 plus adjustment for glucose instead of BMI; model 3 includes all of the variables from model 1 plus adjustment for systolic blood pressure instead of BMI; model 4 includes all of the variables from model 1 plus adjustment for HOMA-IR instead of BMI; and model 5 includes all of the variables from model 1 plus adjustment for hsCRP instead of BMI. CKD, chronic kidney disease; BMI, body mass index; CI, confidence intervals; PR, prevalence ratio. CKD is defined as GFR < 60 ml/min per 1.73 m2 (DOCX) [file pone.0175298.s005.docx]

**S1 Table. Mediation analysis of the associations of sleep duration and subjective sleep quality with CKD and glomerular hyperfiltration among men**

|  | **Sleep duration (hours)** | | | | | ***P* for quadratic trend** | **Subjective sleep quality** | |
| --- | --- | --- | --- | --- | --- | --- | --- | --- |
|  | **≤ 5** | **6** | **7** | **8** | **≥ 9** |  | **Good** | **Poor** |
| **Number** | 36,459 | 85,662 | 80,684 | 32,135 | 6,667 |  | 197,405 | 44,202 |
| **CKD** |  |  |  |  |  |  |  |  |
| **Model 1** | 1.20 (0.94-1.53) | 0.92 (0.74-1.13) | Reference | 0.97 (0.75-1.26) | 1.56 (1.06-2.29) | 0.051 | Reference | 1.06 (0.84-1.32) |
| **Model 2** | 1.22 (0.96-1.55) | 0.93 (0.75-1.14) | Reference | 0.97 (0.75-1.26) | 1.56 (1.06-2.30) | 0.060 | Reference | 1.06 (0.84-1.32) |
| **Model 3** | 1.22 (0.95-1.55) | 0.93 (0.75-1.14) | Reference | 0.94 (0.73-1.23) | 1.52 (1.03-2.24) | 0.105 | Reference | 1.09 (0.87-1.37) |
| **Model 4** | 1.17 (0.91-1.50) | 0.92 (0.74-1.13) | Reference | 0.94 (0.72-1.23) | 1.61 (1.07-2.41) | 0.065 | Reference | 1.08 (0.86-1.36) |
| **Model 5** | 1.16 (0.86-1.57) | 0.89 (0.69-1.15) | Reference | 0.79 (0.56-1.12) | 1.87 (1.15-3.04) | 0.103 | Reference | 1.15 (0.88-1.52) |
| **Hyperfiltration** |  |  |  |  |  |  |  |  |
| **Model 1** | 1.02 (0.94-1.10) | 0.97 (0.92-1.03) | Reference | 1.03 (0.94-1.13) | 1.39 (1.13-1.72) | 0.002 | Reference | 1.09 (1.02-1.17) |
| **Model 2** | 1.00 (0.93-1.08) | 0.97 (0.92-1.03) | Reference | 1.03 (0.94-1.13) | 0.39 (1.13-1.72) | 0.002 | Reference | 1.09 (1.01-1.17) |
| **Model 3** | 1.00 (0.93-1.08) | 0.97 (0.91-1.03) | Reference | 1.03 (0.94-1.13) | 1.31 (1.06-1.63) | 0.006 | Reference | 1.10 (1.02-1.18) |
| **Model 4** | 1.00 (0.93-1.08) | 0.97 (0.91-1.03) | Reference | 1.02 (0.93-1.12) | 1.35 (1.09-1.68) | 0.006 | Reference | 1.09 (1.02-1.17) |
| **Model 5** | 1.02 (0.94-1.11) | 0.97 (0.91-1.04) | Reference | 1.01 (0.92-1.13) | 1.43 (1.12-1.82) | 0.011 | Reference | 1.14 (1.06-1.23) |

^a^ Estimated from multinomial logistic regression models. Multivariable model 1 was adjusted for age, center, year of screening exam, smoking status, alcohol intake, physical activity, marital status, education level, total calorie intake, depression, history of diabetes, history of hypertension, history of cardiovascular disease, and BMI; model 2 includes all of the variables from model 1 plus adjustment for glucose instead of BMI; model 3 includes all of the variables from model 1 plus adjustment for systolic blood pressure instead of BMI; model 4 includes all of the variables from model 1 plus adjustment for HOMA-IR instead of BMI; and model 5 includes all of the variables from model 1 plus adjustment for hsCRP instead of BMI.

CKD, chronic kidney disease; BMI, body mass index; CI, confidence intervals; PR, prevalence ratio.

CKD is defined as GFR < 60 ml/min per 1.73 m^2^
