# Supplementary material for: Sleep duration and quality in relation to chronic kidney disease and glomerular hyperfiltration in healthy men and women
Source: PLoS One. 2017 Apr 19;12(4):e0175298. doi: 10.1371/journal.pone.0175298 (PMC5396878; doi:10.1371/journal.pone.0175298)
Supplement: S3 Table — a Estimated from multinomial logistic regression models. Multivariable model 1 was adjusted for age, center, year of screening exam, smoking status, alcohol intake, physical activity, marital status, education level, total caloric intake, and depression; model 2 includes all of the variables from model 1 plus adjustment for history of diabetes, history of hypertension, and history of cardiovascular disease. CKD, chronic kidney disease; BMI, body mass index; CI, confidence intervals; PR, prevalence ratio. CKD is defined as GFR < 60 ml/min per 1.73 m2 (DOCX) [file pone.0175298.s007.docx]

**S3 Table. Odds ratios^a^ (95% CI) of proteinuria according to sleep duration and subjective sleep quality among men**

|  | **Sleep duration (hours)** | | | | | ***P* for quadratic trend** | **Subjective sleep quality** | |
| --- | --- | --- | --- | --- | --- | --- | --- | --- |
|  | **≤5** | **6** | **7** | **8** | **≥9** |  | **Good** | **Poor** |
| **Number** | 21,081 | 55,249 | 45,242 | 12,533 | 1,528 |  | 115,623 | 20,010 |
| **Proteinuria** |  |  |  |  |  |  |  |  |
| **Cases (%)** | 375 (1.8) | 834 (1.5) | 686 (1.5) | 203 (1.6) | 45 (3.0) |  | 1,757 (1.5) | 386 (1.9) |
| **Crude** | 1.18 (1.04-1.34) | 1.00 (0.90-1.10) | Reference | 1.07 (0.91-1.25) | 1.97 (1.45-2.68) | 0.001 | Reference | 1.27 (1.14-1.42) |
| **Multivariate-adjusted PRs^a^** |  |  |  |  |  |  |  |  |
| **Model 1** | 1.04 (0.92-1.18) | 0.96 (0.87-1.07) | Reference | 1.00 (0.85-1.18) | 1.55 (1.13-2.12) | 0.021 | Reference | 1.15 (1.02-1.29) |
| **Model 2** | 1.02 (0.90-1.16) | 0.96 (0.87-1.07) | Reference | 1.00 (0.85-1.17) | 1.48 (1.07-2.03) | 0.042 | Reference | 1.11 (0.99-1.25) |

^a^ Estimated from multinomial logistic regression models. Multivariable model 1 was adjusted for age, center, year of screening exam, smoking status, alcohol intake, physical activity, marital status, education level, total caloric intake, and depression; model 2 includes all of the variables from model 1 plus adjustment for history of diabetes, history of hypertension, and history of cardiovascular disease.

CKD, chronic kidney disease; BMI, body mass index; CI, confidence intervals; PR, prevalence ratio.

CKD is defined as GFR < 60 ml/min per 1.73 m^2^
