# Supplementary material for: Sleep duration and quality in relation to chronic kidney disease and glomerular hyperfiltration in healthy men and women
Source: PLoS One. 2017 Apr 19;12(4):e0175298. doi: 10.1371/journal.pone.0175298 (PMC5396878; doi:10.1371/journal.pone.0175298)
Supplement: S4 Table — a Estimated from multinomial logistic regression models. Multivariable model 1 was adjusted for age, center, year of screening exam, smoking status, alcohol intake, physical activity, marital status, education level, total caloric intake, and depression; model 2 includes all of the variables from model 1 plus adjustment for history of diabetes, history of hypertension, and history of cardiovascular disease. CKD, chronic kidney disease; BMI, body mass index; CI, confidence intervals; PR, prevalence ratio. CKD is defined as GFR < 60 ml/min per 1.73 m2. (DOCX) [file pone.0175298.s008.docx]

**S4 Table. Odds ratios^a^ (95% CI) of proteinuria according to sleep duration and subjective sleep quality among women**

|  | **Sleep duration (hours)** | | | | | ***P* for quadratic trend** | **Subjective sleep quality** | |
| --- | --- | --- | --- | --- | --- | --- | --- | --- |
|  | **≤5** | **6** | **7** | **8** | **≥9** |  | **Good** | **Poor** |
| **Number** | 15,378 | 30,413 | 35.442 | 19,602 | 5,139 |  | 81,782 | 24,192 |
| **Proteinuria** |  |  |  |  |  |  |  |  |
| **Cases (%)** | 259 (1.7) | 402 (1.3) | 449 (1.3) | 228 (1.2) | 60 (1.2) |  | 1,045 (1.3) | 353 (1.5) |
| **Crude** | 1.34 (1.14-1.56) | 1.04 (0.91-1.20) | Reference | 0.92 (0.78-1.08) | 0.92 (0.70-1.21) | 0.053 | Reference | 1.14 (1.01-1.29) |
| **Multivariate-adjusted PRs^a^** |  |  |  |  |  |  |  |  |
| **Model 1** | 1.20 (1.02-1.40) | 0.95 (0.83-1.09) | Reference | 0.98 (0.83-1.15) | 0.97 (0.74-1.28) | 0.304 | Reference | 1.07 (0.94-1.22) |
| **Model 2** | 1.17 (1.00-1.37) | 0.95 (0.83-1.09) | Reference | 0.97 (0.82-1.14) | 0.95 (0.72-1.25) | 0.711 | Reference | 1.05 (0.92-1.19) |

^a^ Estimated from multinomial logistic regression models. Multivariable model 1 was adjusted for age, center, year of screening exam, smoking status, alcohol intake, physical activity, marital status, education level, total caloric intake, and depression; model 2 includes all of the variables from model 1 plus adjustment for history of diabetes, history of hypertension, and history of cardiovascular disease.

CKD, chronic kidney disease; BMI, body mass index; CI, confidence intervals; PR, prevalence ratio.

CKD is defined as GFR < 60 ml/min per 1.73 m^2^
